# A sleep modulated Channelopathy: a novel CACNA1A pathogenic variant identified in episodic Ataxia type 2 and a potential link to sleep alleviated migraine

**DOI:** 10.1186/s12883-019-1491-3

**Published:** 2019-10-22

**Authors:** Abhimanyu S. Ahuja, Todd D. Rozen, Paldeep S. Atwal

**Affiliations:** 1 Charles E. Schmidt College of Medicine, Florida Atlantic University, Boca Raton, FL 33431 USA; 2 Department of Neurology, Mayo Clinic Florida, Mayo Clinic Jacksonville, 4500 San Pablo Road, Jacksonville, FL 32224 USA; 3 Clinical & Biochemical Geneticist, Atwal Clinic, Jacksonville, FL USA

**Keywords:** Episodic ataxia type 2, Sleep, Channelopathy, P/Q calcium channel, Hemiplegic migraine, Acetazolamide

## Abstract

**Background:**

To describe a patient with sleep alleviated episodic ataxia type 2 with a novel CACNA1A pathogenic variant and provide a possible link to sleep responsive migraine.

**Case presentation:**

A 26-year-old woman with recurrent attacks of dizziness, nausea, vomiting, ataxia and dysarthria presented for a possible diagnosis of vestibular migraine. Unique to her attacks was if she could fall asleep for as little as 15 min the spells would subside. If however she remained awake the attacks would continue unabated. A presumed diagnosis of episodic ataxia type 2 was made and she became attack free on acetazolamide without recurrence. Genetic testing demonstrated a novel pathogenic variant in *CACNA1A* on chromosome 19. This pathogenic variant has not been previously reported in the literature and is suggested to truncate the CACNA1A polypeptide by introducing a premature stop codon.

**Conclusion:**

A case of episodic ataxia type 2 with a novel pathogenic variant in *CACNA1A* is described. Interestingly, the patient’s symptoms would completely alleviate with sleep which suggests a sleep modulated channelopathy. The mechanisms by which sleep could potentially alter this pathogenic variant are hypothesized. A potential link to sleep alleviated migraine is suggested. Further study of this novel pathogenic variant may help us understand not only how sleep can modulate episodic ataxia type 2, but also migraine.

## Background

Episodic Ataxia type 2 (EA-2) is a heritable disorder marked by transient episodes of prolonged ataxia, vertigo, and interictal nystagmus [1]. EA-2 can be traced to pathogenic variants in the *CACNA1A* on chromosome 19. This gene encodes for the main subunit (Ca_v_2.1) of the P/Q type voltage-gated calcium channel [2]. This form of episodic ataxia is acetazolamide responsive, thus patients become symptom free with the carbonic–anhydrase inhibitor.

We report a case of EA-2 with a novel pathogenic variant in *CACNA1A* which, to our knowledge, has not been previously described. Interestingly, the patient’s symptoms would completely alleviate with sleep.

## Case presentation

A 26-year-old woman presented to a headache specialty clinic for a possible diagnosis of vestibular migraine. She was referred by otolaryngology. She was experiencing recurrent bouts of dizziness, lightheadedness, nausea, and ataxia. The episodes started in her late teens and were always stereotyped. The attacks would begin with dizziness and lightheadedness (never vertigo) and eventually progress to the point where she would become nauseous and would vomit. She would then notice she could not walk in a straight line and would need assistance to ambulate. Her hands would become tremulous and she would have trouble writing. She also would develop slurred speech and blurred vision. She would sometimes develop a headache during her spells but this was not consistent and she did not feel the headaches were a dominant part of her syndrome. Uniquely, the only thing that could cause the attacks to subside was sleep. Sleeping just 15 min could relieve her symptoms completely. If she could not find time to sleep the episodes would remain unabated. Thus on work days her bouts could last 12 plus hours in duration. If however she could fall asleep immediately at the onset of an episode it would be gone when she awoke. The spells would occur on average once every 2 weeks with the longest span in between bouts of 6 weeks. She could also experience attacks several days in a row. She did not notice any circannual or diurnal pattern to her spells. The patient reported that stress, anxiety, fatigue, heat, caffeine, and alcohol could trigger her episodes. Her past medical history was significant for episodic migraine without aura and Vitamin B12 deficiency. Family history was significant for Tourette syndrome in her father and a paternal cousin. No familial migraine history was noted, nor did any family member have similar spells. Prior treatment focused on a vertigo syndrome including the use of meclizine and clonazepam without benefit. Neurologic examination in between attacks was normal. She was never examined during a spell as treatment provided at her initial consultation (see below) caused immediate cessation of her episodes. Brain MRI, MRA head and neck, and MR venography were normal.

A possible diagnosis of EA-2 was entertained. She became completely attack free on acetazolamide (250 mg bid) with a 2.5 year follow-up. Exon sequencing of *CACNA1A* revealed a novel heterozygous pathogenic variant in exon 19 denoted c.2496dupC, p.Asn833GlnfsTer1088 (p.Asn833fs). This variant is a duplication of a cytosine at position 2496, which results in a downstream change from asparagine to glutamine at amino acid position 833, with a resultant altered reading frame. This pathogenic variant had not been previously reported in the literature and was suggested to truncate the CACNA1A polypeptide by introducing a premature stop codon. Vestibular migraine was indeed in the differential for this patient. Some patients with this syndrome respond to acetazolamide, but the *CACNA1A* pathogenic variant has not been identified in this distinct migraine population [3, 4].

## Discussion and conclusions

Many different pathogenic variants have been found in *CACNA1A* leading to EA-2. However, the pathogenic variant in our patient has not been previously reported and the alleviation of attacks with sleep is extremely distinctive. This novel variant is defined as pathogenic given the latest American College of Genetics and Genomics guidelines. One prior case of sleep modulated presumed EA-2 has been noted in the literature, but that patient did not have genetic testing to prove the diagnosis [5]. Based on the novel pathogenic variant described in our patient, it is likely the alpha subunit of the Ca^2+^ channel becomes truncated just beyond the synprint site. Previously, haploinsufficiency has been suggested as a mechanism for EA-2, which would result in an approximately 50% reduction of Ca_v_2.1 activity [6]. However, the analysis of Ca_v_2.1^(+/−)^ heterozygous mice, with a 50% reduction of Ca_v_2.1 activity, have shown no clear neurologic abnormalities [6]. Mezghrani et al. [6] utilizing “pulse-chase experiments” revealed that misfolded mutants bind to nascent wild-type Ca_v_ subunits and induce their subsequent degradation, thereby abolishing channel activity.” Therefore, instead of haploinsufficiency, a dominant negative effect could be the cause of EA-2.

The P/Q calcium channels affected in EA-2 are found abundantly in the Purkinje cells of the cerebellum. Walter et al. [7] have suggested that the clinical symptoms of EA-2 are a result of a loss of precision of the pacemaking of these Purkinje cells. Investigations have shown that the precision of Purkinje cell pacemaking is maintained by small conductance (SK) and large conductance (BK) K_Ca_ channels [7]. P/Q channel mutations implicated in EA-2 have been noted to reduce the precision of intrinsic Purkinje cell pacemaking due to fewer K_Ca_ cells being activated with each action potential [ 7]. It is likely that if more K_Ca_ channels are activated with each action potential the precision of pacemaking in mutant Purkinje cells could be restored [7]. The mode of action of carbonic anhydrase inhibitors such as acetazolamide in the treatment of EA-2 is not currently understood. It has been shown that therapeutic concentrations of carbonic anhydrase inhibitors activate BK channels so their therapeutic mode of action could be the recovery of regular pacemaking in Purkinje cells [ 7].

While it is unclear how sleep can potentially modulate this distinct calcium channel mutation, an effect may be coming through K_Ca_ channels. It has been shown that in the suprachiasmatic nucleus BK channel currents and expression are highest at night [8]. It is believed that such activation of BK channels is involved with maintaining the circadian rhythm [8]. If a similar activation of BK channels occurs during sleep in the cerebellum this may be why our patient noticed a relief in her symptoms after sleeping. Increased activation of SK channels may also be involved, though it is unclear how sleep would be associated with this.

Migraine is another episodic disorder that can be modulated by sleep. Migraine can be alleviated only by sleep in some individuals, while sleep can trigger migraine headache in others [9, 10]. Migraine has been recognized as part of the EA-2 clinical spectrum [2]. Familial hemiplegic migraine type 1 is a monogenetic disorder linked to the same *CACNA1A* pathogenic variant as EA-2 [11]. It would be very interesting to note if individuals with sleep alleviated familial hemiplegic migraine have the same pathogenic variant as in our EA-2 patient. Could non familial hemiplegic migraine patients who have predominant sleep alleviated headaches also carry this same pathogenic variant? Orexin is a primary neuro-excitatory hypothalamic sleep regulating neurotransmitter. Orexin A neurons have been found in animal models to project from the hypothalamus to the cerebellum and vestibular complex [12]. Orexin A has been shown to enhance neuronal depolarization in various CNS neurons via voltage gated Ca channels [13, 14]. Thus, could orexin A in some capacity influence sleep responsive EA-2 by altering cerebellar calcium channel function, essentially turning off EA-2 attacks with sleep? More interesting is orexin A’s capacity as a trigeminal nociceptive inhibitor, thus possibly implicating this neurotransmitter in the sleep alleviation of some forms of migraine, especially if our noted novel mutation is present [15]. Further study is warranted.

In conclusion, we have identified a unique subtype of EA-2 which appears to be a sleep modulated channelopathy. EA-2 is an uncommon disorder but should be in the differential of undiagnosed attacks of dizziness and ataxia and/or in patients with possible migrainous vertigo. Further study of this novel pathogenic variant may help us understand not only how sleep can modulate EA-2, but also migraine.

## Data Availability

Case Report-so all available data is within manuscript.

## Abbreviations

EA-2: Episodic ataxia type 2
MRA: Magnetic resonance angiography
MRI: Magnetic resonance imaging
MRV: Magnetic resonance venography
